# The Impact of Striatal Dopaminergic Deficits and Alpha‐Synucleinopathy on Cognitive Function in REM Sleep Behavior Disorder

**DOI:** 10.1002/alz70856_100364

**Published:** 2025-12-25

**Authors:** Vijaya L Reddy, Jesse M Cedarbaum, John Seibyl, Kara Mead, Erika Renkl, Sapna Patel, Samantha Esposito, Sophia Moret, Millie Lawrence, Raina Vin, Carolyn A Fredericks

**Affiliations:** ^1^ Yale University School of Medicine, New Haven, CT, USA

## Abstract

**Background:**

Presynaptic protein α‐synuclein (αSyn) plays a role in Alzheimer's disease (AD) and Parkinson's disease (PD) with evidence suggesting αSyn pathology preceding and driving striatal dopaminergic neurodegeneration in PD. Elevated cerebrospinal fluid (CSF) αSyn seeding activity and reduced striatal dopaminergic activity are associated with cognitive decline in synucleinopathies including PD and Dementia with Lewy bodies (DLB). This study aims to evaluate the understudied relationship between CSF αSyn aggregation and striatal dopaminergic deficits and their impact on cognitive performance in REM sleep behavior disorder (RBD), a known preclinical stage of synucleinopathy.

**Method:**

Thirty‐five RBD participants (aged 51 to 80, MoCA >23) underwent a 123‐I ioflupane SPECT dopamine transporter (DaT) scan, lumbar puncture, and the Montreal Cognitive Assessment (MoCA). A subset (*n* = 9) completed additional cognitive tests, including the Hooper Visual Organization Task, Trail‐Making tasks, Forward and Backward Digit Span tasks, and the Rey Auditory Verbal Learning Test.

**Result:**

CSF αSyn analysis identified 25 αSyn‐positive and 10 αSyn‐negative participants. Semi‐quantitative analysis categorized participants into DaT‐positive (striatal dopaminergic activity <65% of age‐expected levels, *n* = 7), DaT‐intermediate (65‐80%, *n* = 11), and DaT‐negative (>80%, *n* = 17). αSyn positivity was highest in DaT‐positive (86%) and intermediate (82%) groups, decreasing to 59% in DaT‐negative participants.

Although not statistically significant, DaT‐positive participants exhibited lower visuospatial/executive function, working memory index, and total MoCA scores compared to DaT‐negative participants. Among DaT‐intermediate participants, cognitive performance varied, with scores exceeding those of DaT‐negatives on certain tasks and falling below DaT‐positives on others. Interestingly, αSyn‐positive participants showed significantly higher working memory index scores (12.52 ± 2.55 vs. 10.10 ± 3.98, *p* = 0.05) and overall MoCA scores (26.60 ± 3.01 vs. 24.20 ± 3.64, *p* = 0.05) compared to αSyn‐negative participants. This trend was consistent in the subset undergoing detailed cognitive testing.

**Conclusion:**

In RBD, DaT positivity appeared associated with cognitive impairment, while αSyn positivity unexpectedly correlated with better cognitive performance in our small sample. Participant characteristics may limit the findings’ generalizability and biases the sample toward those on a PD trajectory or far from phenoconversion. Application of these potential biomarkers for phenoconversion to clinical synucleinopathy warrants further investigation.

